# Supplementary material for: Pain as a Protective Factor for Alzheimer Disease in Patients with Cancer
Source: Cancers (Basel). 2022 Dec 30;15(1):248. doi: 10.3390/cancers15010248 (PMC9818585; doi:10.3390/cancers15010248)
Supplement: Supplementary file 1 [file cancers-15-00248-s001.zip › cancers-2076811-supplementary.pdf]

Supplementary Materials

Figure S1. Proportion of males and females in case and control group.

Figure S2. Proportion of different races in case and control group.

Figure S3. Proportion of different cancer sites in case and control group.

Figure S4. Hypothesis of the mechanism of pain action in AD and cancer.

Table S1. Baseline characteristics of cases and controls, stratified by race.

Table S2. Risk factors for AD in patients with cancer, stratified by race.

Table S3. Baseline characteristics of cases and controls, stratified by cancer site.

Table S4. Risk factors for AD in patients with cancer, stratified by cancer site.

Table S1. Baseline characteristics of cases and controls, stratified by race.

	White			Asian/Pacific Islander			Black		
**Variable**	**Case group, No. (%) (n=23355)**	**Control group, No. (%) (n=106957)**	***p-value***	**Case group, No. (%) (n=1217)**	**Control group, No. (%) (n=13254)**	***p-value***	**Case group, No. (%) (n=891)**	**Control group, No. (%) (n=7223)**	***p-value***
Age at cancer diagnosis, mean (SD), y	71.91 (9.75)	71.99 (9.78)	0.25[a]	72.77 (9.92)	70.88 (9.79)	<0.001[a]	70.85 (9.87)	69.22 (9.62)	<0.001[a]
Sex									
Male	8037 (34.4)	50868 (47.6)	<0.001[b]	437 (35.9)	6996 (52.8)	<0.001[b]	353 (39.6)	3728 (51.6)	<0.001[b]
Female	15318 (65.6)	56089 (52.4)		780 (64.1)	6258 (47.2)		538 (60.4)	3495 (48.4)	
Pain rating[c]									
I	1950 (8.3)	6834 (6.4)	<0.001[b]	86 (7.1)	871 (6.6)	0.51[b]	80 (9)	454 (6.3)	0.002[b]
II	21405 (91.7)	100123 (93.6)		1131 (92.9)	12383 (93.4)		811 (91)	6769 (93.7)	
Total number of in situ/malignant tumors	1.17 (0.45)	1.21 (0.50)	<0.001[a]	1.18 (0.44)	1.19 (0.46)	0.37[a]	1.13 (0.39)	1.18 (0.46)	<0.001[a]
Total number of benign/borderline tumors	0 (0.07)	0 (0.04)	<0.001[a]	0 (0.057)	0 (0.060)	0.85[a]	0.01 (0.088)	0 (0.056)	>0.99[a]
Cancer site									
Bone and Joint	10 (0)	52 (0)	<0.001[b]	0 (0)	9 (0.1)	<0.001[b]	0 (0)	4 (0.1)	<0.001[b]
Breast	7698 (33)	25496 (23.8)		377 (31)	2874 (21.7)		298 (33.5)	1629 (22.6)	
Digestive	4476 (19.2)	25199 (23.6)		345 (28.3)	4045 (30.5)		208 (23.4)	1716 (23.8)	
Endocrine	224 (1)	803 (0.8)		16 (1.3)	276 (2.1)		3 (0.3)	75 (1)	
Hematological	259 (1.1)	1642 (1.5)		12 (1)	202 (1.5)		8 (0.9)	79 (1.1)	
Kaposi Sarcoma	16 (0.1)	162 (0.2)		1 (0.1)	9 (0.1)		0 (0)	10 (0.1)	
Mesothelioma	1 (0)	13 (0)		0 (0)	2 (0)		1 (0.1)	0 (0)	
Miscellaneous	3 (0)	5 (0)		0 (0)	2 (0)		0 (0)	0 (0)	
Respiratory	478 (2)	5803 (5.4)		27 (2.2)	655 (4.9)		17 (1.9)	404 (5.6)	

Skin and Soft Tissue	1393 (6)	5304 (5)		22 (1.8)	159 (1.2)		9 (1)	87 (1.2)	
Urogenital	8797 (37.7)	42478 (39.7)		417 (34.3)	5021 (37.9)		346 (38.9)	3219 (44.6)	
Radiation therapy									
Yes	5888 (25.2)	26061 (24.4)	0.007[b]	336 (27.6)	3969 (29.9)	0.09[b]	271 (30.4)	2277 (31.5)	0.50[b]
No	17467 (74.8)	80896 (75.6)		881 (72.4)	9285 (70.1)		620 (69.6)	4946 (68.5)	
Cancer-directed surgery									
Yes	21376 (91.5)	95543 (89.3)	<0.001[b]	1073 (88.2)	11365 (85.7)	0.02[b]	774 (86.9)	5980 (82.8)	0.002[b]
No	1979 (8.5)	11414 (10.7)		144 (11.8)	1889 (14.3)		117 (13.1)	1243 (17.2)	
Chemotherapy									
Yes	1669 (7.1)	6851 (6.4)	<0.001[b]	104 (8.5)	1208 (9.1)	0.51[b]	80 (9)	548 (7.6)	0.14[b]
No/Unknown	21686 (92.9)	100106 (93.6)		1113 (91.5)	12046 (90.9)		811 (91)	6675 (92.4)	
Survival years after the first tumor diagnosis	15.58 (8.86)	11.64 (8.15)	<0.001[a]	16.51 (9.10)	12.63 (8.34)	<0.001[a]	15.52 (8.64)	11.15 (7.84)	<0.001[a]

[a] *p-value* by independent t-test. [b] *p-value* by X^2 test.
[c] Pain rating I indicates in situ behavior code; pain rating II, malignant behavior code, with survival years>1.

Table S2. Risk factors for AD in patients with cancer, stratified by race.

	White (23355 case; 106957 control)				Asian/Pacific Islander (1217 case; 13254 control)				Black (891 case; 7223 control)			
**Variable**	**OR (95% CI)[a]**	***p-value***	**Adjusted OR (95% CI)[b]**	***p-value***	**OR (95% CI)[a]**	***p-value***	**Adjusted OR (95% CI)[b]**	***p-value***	**OR (95% CI)[a]**	***p-value***	**Adjusted OR (95% CI)[b]**	***p-value***
Age at cancer diagnosis, mean, y	NA	NA	NA	NA	1.091 (1.082-1.101)	<.001	1.092 (1.083-1.102)	<.001	1.075 (1.065-1.086)	<.001	1.076 (1.066-1.086)	<.001
Sex												
Male	1 [Reference]		1 [Reference]		1 [Reference]		1 [Reference]		1 [Reference]		1 [Reference]	
Female	1.434 (1.383-1.487)	<0.001	1.435 (1.384-1.489)	<0.001	1.696 (1.456-1.976)	<0.001	1.701 (1.499-1.929)	<0.001	1.083 (0.893-1.313)	0.42	NA	NA
Pain rating												
I	1 [Reference]		1 [Reference]		1 [Reference]		1 [Reference]		1 [Reference]		1 [Reference]	
II	0.861 (0.815-0.910)	<0.001	0.862 (0.816-0.911)	<0.001	NA	NA	NA	NA	0.879 (0.672-1.150)	0.35	NA	NA
Total number of in situ/malignant tumors	0.751 (0.727-0.775)	<0.001	0.751 (0.727-0.775)	<0.001	NA	NA	NA	NA	0.641 (0.530-0.776)	<0.001	0.641 (0.530-0.775)	<0.001
Total number of benign/borderline tumors	2.222 (1.727-2.857)	<0.001	2.224 (1.729-2.860)	<0.001	NA	NA	NA	NA	NA	NA	NA	NA
Cancer site												
Bone and Joint	0.795 (0.398-1.586)	0.52	0.793 (0.398-1.583)	0.51	0	>0.99	NA	NA	0	>0.99	0	>0.99
Breast	1.107 (1.062-1.155)	<0.001	1.110 (1.064-1.157)	<0.001	1.079 (0.898-1.296)	0.42	NA	NA	1.351 (1.074-1.700)	0.01	1.436 (1.207-1.708)	<0.001
Digestive	0.849 (0.814-0.886)	<0.001	0.848 (0.813-0.884)	<0.001	0.914 (0.774-1.079)	0.29	NA	NA	0.965 (0.783-1.188)	0.74	0.983 (0.813-1.188)	0.86
Endocrine	0.985 (0.844-1.150)	0.85	0.986 (0.845-1.152)	0.86	0.633 (0.372-1.078)	0.09	NA	NA	0.465 (0.143-1.511)	0.20	0.474 (0.146-1.532)	0.21
Hematological	0.675 (0.588-0.776)	<0.001	0.675 (0.588-0.775)	<0.001	0.716 (0.390-1.314)	0.28	NA	NA	1.102 (0.516-2.356)	0.80	1.127 (0.528-2.404)	0.76
Kaposi Sarcoma	0.550 (0.327-0.923)	0.02	0.550 (0.328-0.923)	0.02	1.319 (0.160-10.874)	0.80	NA	NA	0	>0.99	0	>0.99

Mesothelioma	0.362 (0.046-2.822)	0.33	0.361 (0.046-2.817)	0.33	0	>0.99	NA	NA	4558822632	1	4360511137	1
Miscellaneous	1.747 (0.388-7.869)	0.47	1.741 (0.387-7.841)	0.47	0	>0.99	NA	NA	NA	NA	NA	NA
Respiratory	0.438 (0.398-0.483)	<0.001	0.438 (0.397-0.483)	<0.001	0.719 (0.478-1.081)	0.11	NA	NA	0.567 (0.341-0.943)	0.03	0.575 (0.346-0.954)	0.03
Skin and Soft Tissue	1.208 (1.129-1.291)	<0.001	1.205 (1.127-1.289)	<0.001	1.536 (0.952-2.476)	0.08	NA	NA	0.643 (0.312-1.325)	0.23	0.658 (0.321-1.348)	0.26
Urogenital	1 [Reference]		1 [Reference]		1 [Reference]		1 [Reference]		1 [Reference]		1 [Reference]	
Radiation therapy												
Yes	1.018 (0.977-1.061)	0.39	NA	NA	NA	NA	NA	NA	NA	NA	NA	NA
No	1 [Reference]		1 [Reference]		1 [Reference]		1 [Reference]		1 [Reference]		1 [Reference]	
Cancer-directed surgery												
Yes	0.915 (0.857-0.976)	0.007	0.901 (0.853-0.952)	<0.001	0.836 (0.677-1.032)	0.10	NA	NA	0.943 (0.746-1.192)	0.62	NA	NA
No	1 [Reference]		1 [Reference]		1 [Reference]		1 [Reference]		1 [Reference]		1 [Reference]	
Chemotherapy												
Yes	1.228 (1.158-1.303)	<0.001	1.232 (1.161-1.306)	<0.001	NA	NA	NA	NA	NA	NA	NA	NA
No	1 [Reference]		1 [Reference]		1 [Reference]		1 [Reference]		1 [Reference]		1 [Reference]	
Survival years after the first tumor diagnosis	1.050 (1.048-1.052)	<0.001	1.050 (1.048-1.052)	<0.001	1.118 (1.107-1.129)	<0.001	1.118 (1.107-1.129)	<0.001	1.120 (1.108-1.133)	<0.001	1.121 (1.108-1.133)	<0.001

Abbreviations: OR = odds ratio; CI = confidence interval; NA = not applicable.

[a] The ORs are adjusted for age at diagnosis (>20y), age at death (>65y), and cancer categories by design, and are calculated by univariable logistic regression models.

[b] The adjusted OR are adjusted for other significant variables apart from the one in very row, and are calculated by multivariable logistic regression models.

Table S3. Baseline characteristics of cases and controls, stratified by cancer site.

	**Breast**			**Digestive**			**Skin and Soft Tissue**			**Urogenital**		
**Variable**	**Case group, No. (%) (n=8390)**	**Control group, No. (%) (n=30030)**	***p-value***	**Case group, No. (%) (n=5037)**	**Control group, No. (%) (n=30987)**	***p-value***	**Case group, No. (%) (n=1428)**	**Control group, No. (%) (n=5557)**	***p-value***	**Case group, No. (%) (n=9578)**	**Control group, No. (%) (n=50770)**	***p-value***
Age at cancer diagnosis, mean (SD), y	71.84 (10.21)	71.66 (10.49)	0.17[a]	73.76 (9.51)	73.50 (9.48)	0.08[a]	72.52 (11.67)	71.26 (11.36)	<0.001[a]	71.06 (8.95)	71.18 (9.21)	0.21[a]
Sex												
Male	36 (0.4)	304 (1)	<0.001[b]	1776 (35.3)	16280 (52.5)	<0.001[b]	606 (42.4)	3314 (59.6)	<0.001[b]	6048 (63.1)	36167 (71.2)	<0.001[b]
Female	8354 (99.6)	29726 (99)		3261 (64.7)	14707 (47.5)		822 (57.6)	2243 (40.4)		3530 (36.9)	14603 (28.8)	
Race												
White	7698 (91.8)	25496 (84.9)	<0.001[b]	4476 (88.9)	25199 (81.3)	<0.001[b]	1393 (97.5)	5304 (95.4)	0.001[b]	8797 (91.8)	42478 (83.7)	<0.001[b]
Asian/Pacific Islander	377 (4.5)	2874 (9.6)		345 (6.8)	4045 (13.1)		22 (1.5)	159 (2.9)		417 (4.4)	5021 (9.9)	
Black	298 (3.6)	1629 (5.4)		208 (4.1)	1716 (5.5)		9 (0.6)	87 (1.6)		347 (3.6)	3219 (6.3)	
American Indian/Alaska Native	17 (0.2)	31 (0.1)		8 (0.2)	27 (0.1)		4 (0.3)	7 (0.1)		17 (0.2)	52 (0.1)	
Pain rating												
I	1172(14)	3428 (11.4)	<0.001[b]	372 (7.4)	2636 (8.5)	0.008[b]	390 (27.3)	1317 (23.7)	0.005[b]	183 (1.9)	775 (1.5)	0.006[b]

II	7218 (86)	26602 (88.6)		4665 (92.6)	28351 (91.5)		1038 (72.7)	4240 (76.3)		9395 (98.1)	49995 (98.5)	
Total number of in situ/malignant tumors	1.18 (0.448)	1.21 (0.490)	<0.001[a]	1.18 (0.447)	1.21 (0.497)	<0.001[a]	1.25 (0.575)	1.35 (0.755)	<0.001[a]	1.15 (0.422)	1.18 (0.456)	<0.001[a]
Total number of benign/borderline tumors	0.01 (0.075)	0 (0.050)	0.001[a]	0 (0.056)	0 (0.038)	0.10[a]	0 (0.065)	0 (0.064)	0.55[a]	0 (0.063)	0 (0.041)	<0.001[a]
Radiation therapy												
Yes	2758 (32.9)	9252 (30.8)	<0.001[b]	363 (7.2)	2342 (7.6)	0.38[b]	57 (4)	271 (4.9)	0.16[b]	2995 (31.3)	16708 （32.9）	0.002[b]
No	5632 (67.1)	20778 (69.2)		4674 (92.8)	28645 (92.4)		1371 (96)	5286 (95.1)		6583 (68.7)	34062 （67.1）	
Cancer-directed surgery												
Yes	8350 (99.5)	29787 (99.2)	0.002[b]	4967 (98.6)	30363 (98)	0.003[b]	1427 (99.9)	5534 (99.6)	0.048[b]	7651 （79.9）	39583 (78)	<0.001[b]
No	40 (0.5)	243 (0.8)		70 (1.4)	624 (2)		1(0.1)	23 (0.4)		1927 (20.1)	11187 (22)	
Chemotherapy												
Yes	636 (7.6)	2129 (7.1)	0.12[b]	655 (13)	3030 (9.8)	<0.001[b]	12 (0.8)	69 (1.2)	0.21[b]	340 (3.5)	1669 (3.3)	0.19[b]
No/Unknown	7754 (92.4)	27901 (92.9)		4382 (87)	27957 (90.2)		1416 (99.2)	5488 (98.8)		9238 (96.5)	49101 (96.7)	
Survival years after the first tumor diagnosis	16.17 (8.91)	12.94 (8.59)	<.001[a]	14.73 (8.60)	10.86 (7.73)	<0.001[a]	14.79 (10.04)	12.73 (9.00)	<0.001[a]	15.84 (8.66)	11.69 (7.93)	<0.001[a]

[a] *p-value* by independent t-test.
[b] *p-value* by X^2 test.
[c] Pain rating I indicates in situ behavior code; pain rating II, malignant behavior code, with survival years>1.

Table S4. Risk factors for AD in patients with cancer, stratified by cancer site.

	Breast cancer (8390 case; 30030 control)				Digestive cancer (5037 case; 30987 control)				Skin and Soft Tissue cancer (1428 case; 5557 control)				Urogenital cancer (9578 case; 50770 control)			
**Variable**	**OR (95% CI)**	***p-value***	**Adjusted OR (95% CI)**	***p-value***	**OR (95% CI)**	***p-value***	**Adjusted OR (95% CI)**	***p-value***	**OR (95%CI)**	***p-value***	**Adjusted OR (95% CI)**	***p-value***	**OR (95% CI)**	***p-value***	**Adjusted OR (95% CI)**	***p-value***
Age at cancer diagnosis, mean, y	NA	NA	NA	NA	NA	NA	NA	NA	1.050 (1.042-1.059)	<0.001	1.050 (1.042-1.059)	<0.001	NA	NA	NA	NA
Sex																
Male	1 [Reference]		1 [Reference]		1 [Reference]		1 [Reference]		1 [Reference]		1 [Reference]		1 [Reference]		1 [Reference]	
Female	2.071 (1.459-2.941)	<0.001	2.071 (1.459-2.941)	<0.001	1.937 (1.818-2.064)	<0.001	1.937 (1.818-2.064)	<0.001	1.715 (1.518-1.936)	<0.001	1.715 (1.519-1.937)	<0.001	1.121 (1.065-1.180)	<0.001	1.116 (1.060-1.174)	<0.001
Race																
White	1 [Reference]		1 [Reference]		1 [Reference]		1 [Reference]		1 [Reference]		1 [Reference]		1 [Reference]		1 [Reference]	
Asian/Pacific Islander	0.394 (0.352-0.440)	<0.001	0.394 (0.352-0.440)	<0.001	0.461 (0.410-0.518)	<0.001	0.461 (0.410-0.518)	<0.001	0.532 (0.336-0.843)	0.007	0.524 (0.331-0.829)	0.006	0.382 (0.344-0.424)	<0.001	0.382 (0.345-0.424)	<0.001
Black	0.604 (0.531-0.686)	<0.001	0.604 (0.531-0.686)	<0.001	0.660 (0.568-0.767)	<0.001	0.660 (0.568-0.767)	<0.001	0.358 (0.177-0.722)	0.004	0.359 (0.178-0.724)	0.004	0.542 (0.483-0.607)	<0.001	0.541 (0.483-0.607)	<0.001
American Indian/Alaska Native	1.805 (0.988-3.297)	0.06	1.805 (0.988-3.297)	>0.99	1.682 (0.752-3.762)	0.206	1.682 (0.752-3.762)	0.206	2.769 (0.750-10.223)	0.13	2.779 (0.753-10.261)	0.13	1.642 (0.939-2.871)	0.08	1.645 (0.941-2.877)	0.081
Pain rating																
I	1 [Reference]		1 [Reference]		1 [Reference]		1 [Reference]		1 [Reference]		1 [Reference]		1 [Reference]		1 [Reference]	
II	0.772 (0.718-0.831)	<0.001	0.772 (0.718-0.831)	<0.001	1.130 (1.006-1.270)	0.04	1.130 (1.006-1.270)	0.04	0.818 (0.713-0.938)	0.004	0.815 (0.710-0.934)	0.003	1.142 (0.962-1.355)	0.13	NA	NA

Total number of in situ/malignant tumors	0.738 (0.699-0.780)	<0.001	0.738 (0.699-0.780)	<0.001	0.786 (0.734-0.840)	<0.001	0.786 (0.734-0.840)	<0.001	0.761 (0.688-0.842)	<0.001	0.762 (0.689-0.843)	<0.001	0.733 (0.695-0.774)	<0.001	0.733 (0.695-0.774)	<0.001
Total number of benign/borderline tumors	2.174 (1.497-3.158)	<0.001	2.174 (1.497-3.158)	<0.001	NA	NA	NA	NA	NA	NA	NA	NA	2.283 (1.546-3.371)	<0.001	2.285 (1.547-3.374)	<0.001
Radiation therapy																
Yes	1.164 (1.104-1.228)	<0.001	1.164 (1.104-1.228)	<0.001	NA	NA	NA	NA	NA	NA	NA	NA	0.843 (0.784-0.907)	<0.001	0.847 (0.788-0.911)	<0.001
No	1 [Reference]		1 [Reference]		1 [Reference]		1 [Reference]		1 [Reference]		1 [Reference]		1 [Reference]		1 [Reference]	
Cancer-directed surgery																
Yes	1.775 (1.259-2.501)	0.001	1.775 (1.259-2.501)	0.001	1.499 (1.154-1.947)	0.002	1.499 (1.154-1.947)	0.002	4.844 (0.644-36.437)	0.13	NA	NA	0.778 (0.713-0.849)	<0.001	0.780 (0.715-0.851)	<0.001
No	1 [Reference]		1 [Reference]		1 [Reference]		1 [Reference]		1 [Reference]		1 [Reference]		1 [Reference]		1 [Reference]	
Chemotherapy																
Yes	NA	NA	NA	NA	1.596 (1.449-1.758)	<0.001	1.596 (1.449-1.758)	<0.001	NA	NA	NA	NA	NA	NA	NA	NA
No	1 [Reference]		1 [Reference]		1 [Reference]		1 [Reference]		1 [Reference]		1 [Reference]		1 [Reference]		1 [Reference]	
Survival years after the first tumor diagnosis	1.045 (1.042-1.048)	<0.001	1.045 (1.042-1.048)	<0.001	1.059 (1.055-1.063)	<0.001	1.059 (1.055-1.063)	<0.001	1.073 (1.062-1.083)	<0.001	1.073 (1.062-1.083)	<0.001	1.060 (1.057-1.063)	<0.001	1.060 (1.057-1.063)	<0.001

Abbreviations: OR = odds ratio; CI = confidence interval; NA = not applicable.

[a] The ORs are adjusted for age at diagnosis (>20y), age at death (>65y), and cancer categories by design, and are calculated by univariable logistic regression models.

[b] The adjusted OR are adjusted for other significant variables apart from the one in very row, and are calculated by multivariable logistic regression models.

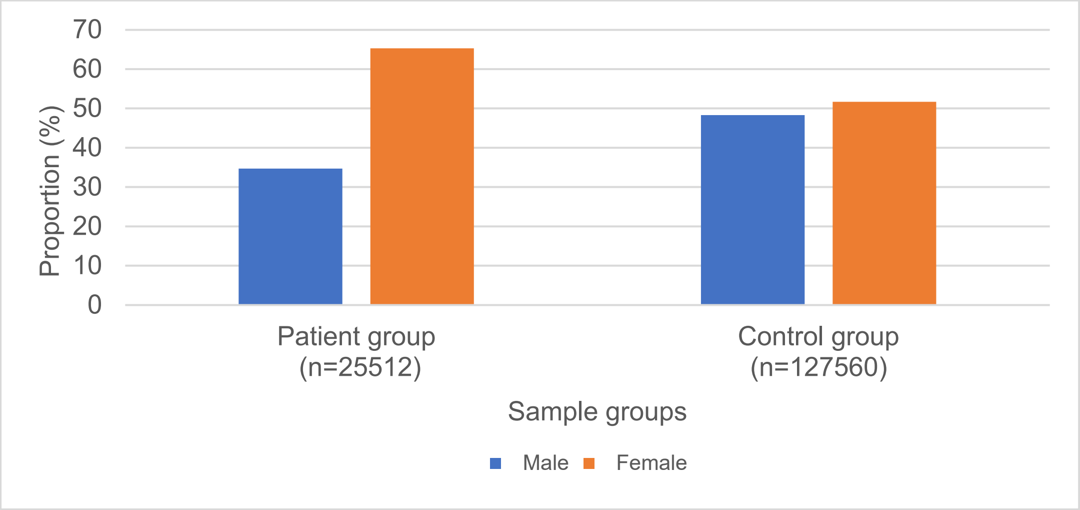

Figure S1. Proportion of males and females in case and control group.

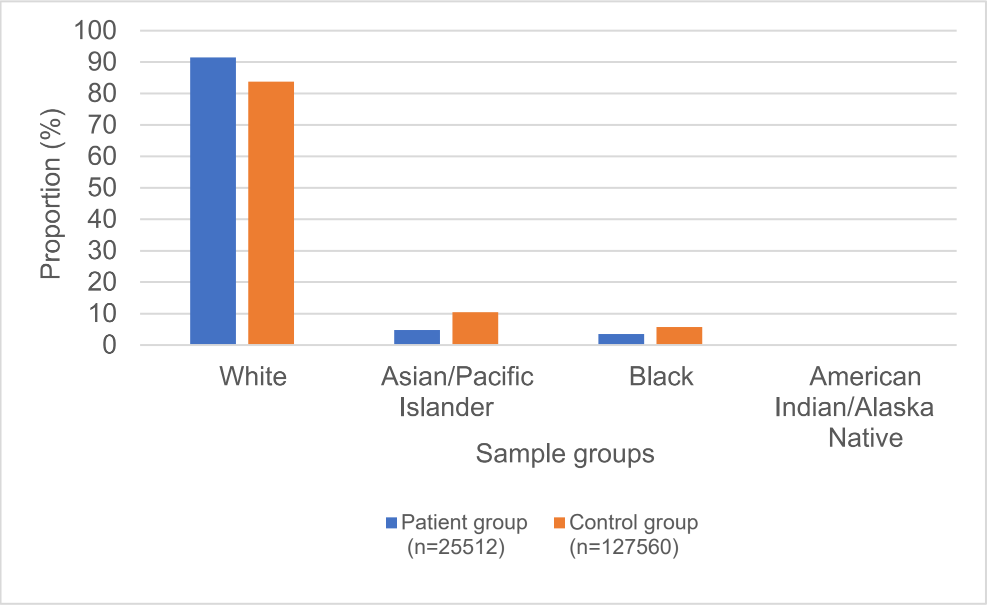

Figure S2. Proportion of different races in case and control group.

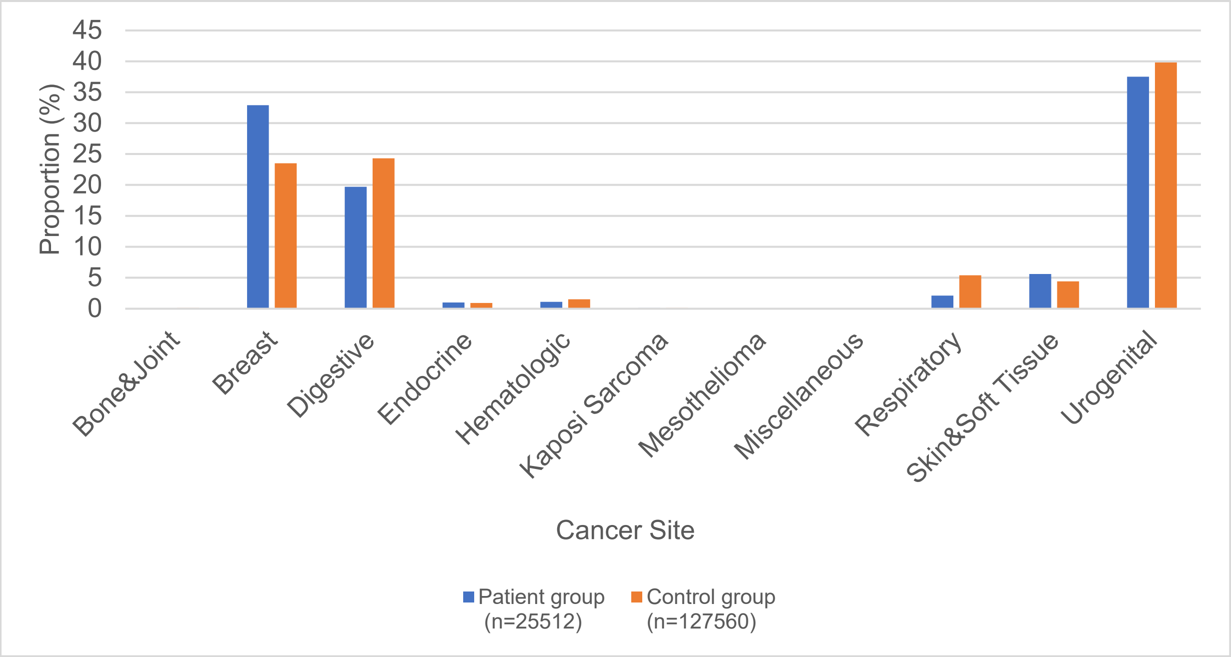

Figure S3. Proportion of different cancer sites in case and control group.

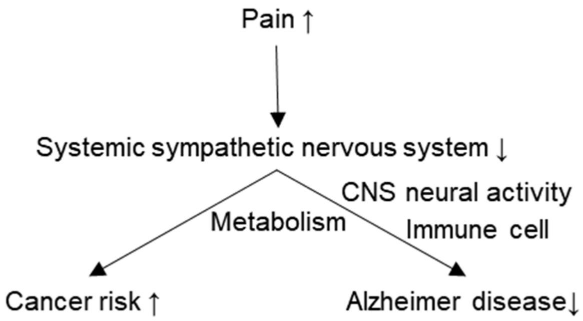

Figure S4. Hypothesis of the mechanism of pain action in AD and cancer.
